# Data supporting the inability of indomethacin to induce autophagy in U251 glioma cells

**DOI:** 10.1016/j.dib.2017.02.012

**Published:** 2017-02-10

**Authors:** Aleksandar Pantovic, Katarina Arsikin, Milica Kosic, Biljana Ristic, Vladimir Trajkovic, Ljubica Harhaji-Trajkovic

**Affiliations:** a Neurology Clinic, Military Medical Academy, Belgrade, Serbia; b Institute of Microbiology and Immunology, School of Medicine, University of Belgrade, Dr. Subotica 1, 11000 Belgrade, Serbia; *c* Institute for Biological Research "Sinisa Stankovic", University of Belgrade, Despot Stefan Blvd. 142, 11000 Belgrade, Serbia

**Keywords:** Indomethacin, Glioma, Autophagy, LC3, Beclin-1

## Abstract

Autophagy, a catabolic process involving intracellular degradation of unnecessary or dysfunctional cellular components through the lysosomal machinery, could act as a prosurvival, as well as a cytotoxic mechanism (Parzych and Klionsky, 2014) [1]. Cyclooxygenase inhibitor indomethacin inhibits proliferation of glioma cells, and has been reported to reduce the activity of the main autophagy repressor mammalian target of rapamycin (mTOR) (Pantovic et al., 2016) [2]. Here we investigated the ability of indomethacin to induce autophagy in U251 human glioma cells. We assessed the influence of indomethacin on intracellular acidification, expression of proautophagic protein beclin-1, and conversion of microtubule-associated protein light chain 3-I (LC3-I) to autophagosome-associated LC3-II, in the presence or absence of lysosomal inhibitors. The effect of genetic and pharmacological downregulation of autophagy on the cytotoxicity of indomethacin was also evaluated. The interpretation of these data can be found in “In vitro antiglioma action of indomethacin is mediated via AMP-activated protein kinase/mTOR complex 1 signaling pathway” (Pantovic et al., 2016; doi:10.1016/j.biocel.2016.12.007) [2].

**Specifications Table**

**Table:** 

Subject area	*Biology*
More specific subject area	*Glioma, autophagy, indomethacin*
Type of data	*Figure*
How data was acquired	*Flow cytometry, immunoblot, siRNA transfection, and MTT viability test*
Data format	*Analyzed*
Experimental factors	*U251 glioma cells were treated with indomethacin and bafilomycin A1, chloroquine, wortmannin, or ammonium chloride, or transfected with LC3, beclin-1, or control siRNA before treatment with indomethacin*
Experimental features	*After indomethacin treatment viability of U251 cells was determined, or the cells were prepared for the flow cytometric and immunoblot assessment of autophagy markers*
Data source location	*School of Medicine, Belgrade, Serbia*
Data accessibility	*All data are provided with this article*

**Value of the data**

• The data provide the assessment of autophagy-inducing ability of indomethacin in glioma cells.
• The data provide the assessment of the effect of pharmacological and genetic inhibition of autophagy on the cytotoxic action of indomethacin.
• These data might be valuable for researchers interested in autophagy-modulating effects of cyclooxygenase inhibitors.
• These data might be valuable for researchers interested in anticancer and antiglioma effects of indomethacin.

## 1 Data

**Fig. 1:**
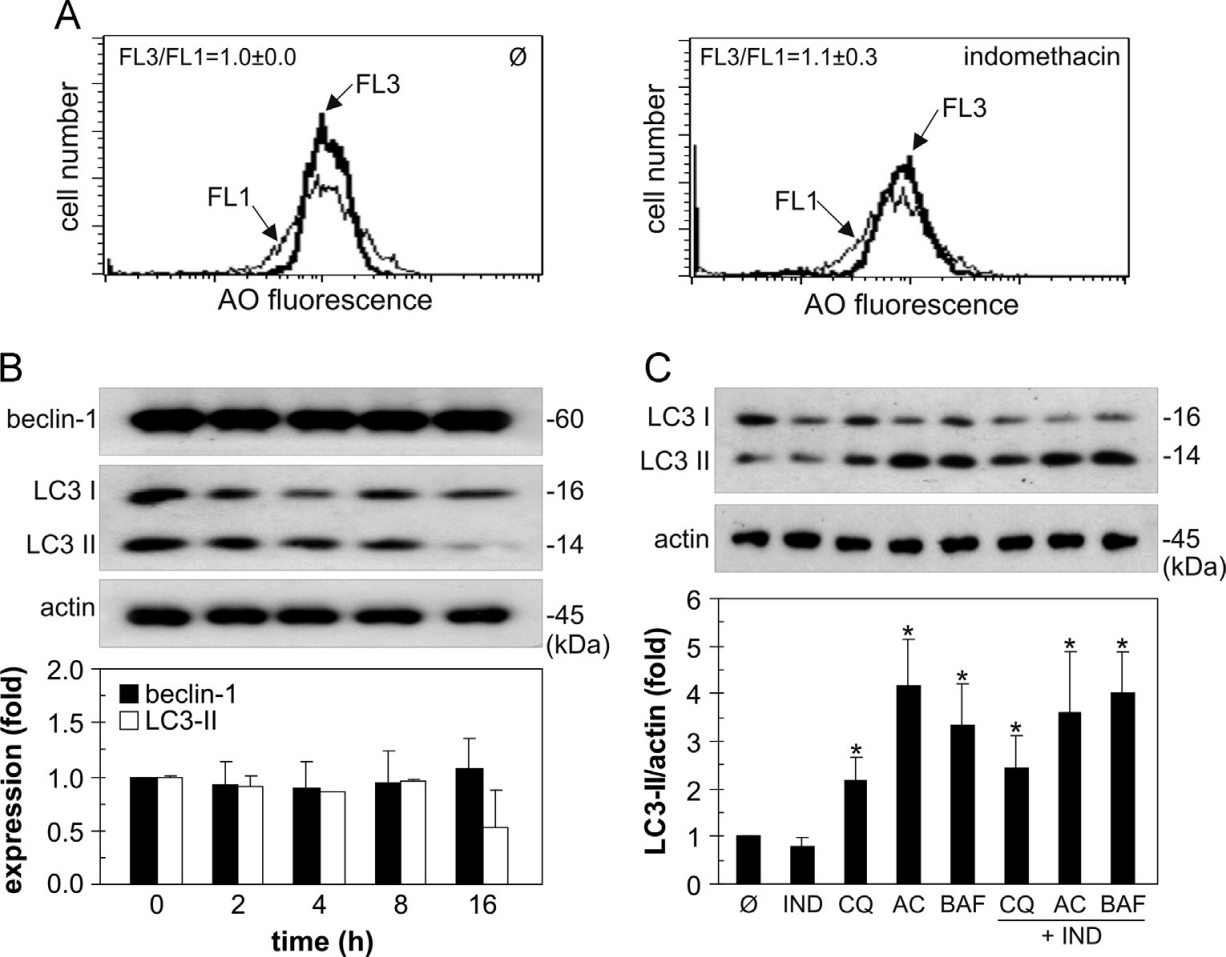
The effect of indomethacin on the expression of autophagy markers in U251 glioma cells. (A) U251 cells were incubated with indomethacin (250 µM) for 24 h. The intracellular acidification was determined by flow cytometry as a ratio of acridine orange (AO) red/green (FL3/FL1) mean fluorescence intensity, and the representative flow cytometry histograms of three independent experiments are shown. (B) U251 cells were incubated with indomethacin (250 µM) for the indicated time periods, and beclin-1 and LC3 levels were analyzed by immunoblotting. (C) Autophagic flux was assessed by examining LC3 conversion in U251 cells incubated with indomethacin (250 µM) for 8 h, in the presence or absence or proteolysis inhibitors chloroquine (CQ; 20 µM), ammonium chloride (AC; 20 mM), or bafilomycin A1 (BAF; 20 nm). (B, C) The representative blots are shown, and the data are presented as mean±SD values of three independent experiments (*p<0.05 compared to untreated cells).

**Fig. 2:**
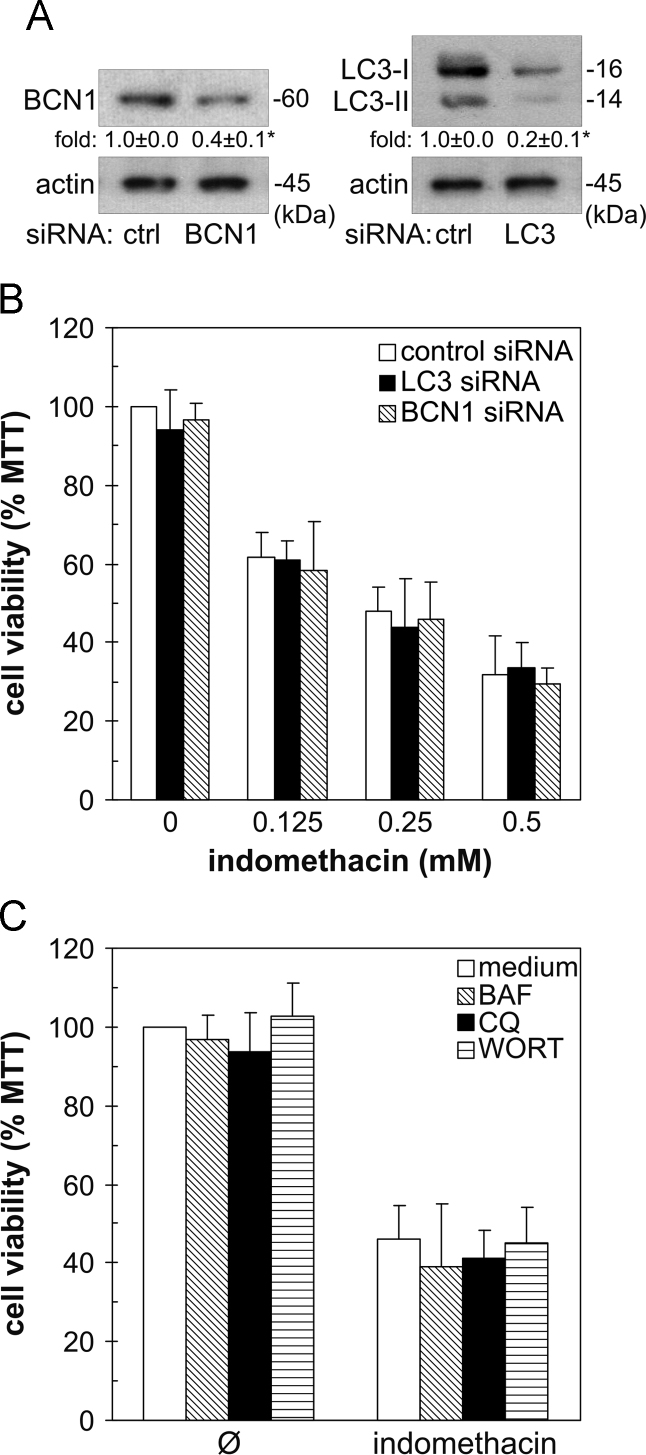
The effect of genetic and pharmacological inhibition of autophagy on indomethacin cytotoxicity. (A, B) U251 cells were transfected with control, beclin (BCN1), or LC3 siRNA. The knockdown efficiency was assessed by immunoblot (A). The siRNA-transfected cells were incubated with different concentrations of indomethacin for 24 h, and the cell viability was assessed by MTT assay (B). (C) U251 cells were treated for 24 h with indomethacin (250 µM) in the presence or absence of autophagy inhibitors bafilomycin A1 (BAF; 20 nM), chloroquine (CQ; 20 µM), or wortmannin (WORT; 200 nM), and cell viability was analyzed by MTT assay. (A-C) The data are presented as mean±SD values of at least three independent experiments (*p<0.05).

To investigate the ability of indomethacin to induce autophagy in U251 glioma cells, we assessed its influence on intracellular acidification, expression of beclin-1, and conversion of microtubule-associated protein light chain 3 (LC3) as autophagy hallmarks [1]. Flow cytometric analysis of pH-sensitive acridine orange fluorescence revealed that indomethacin failed to increase intracellular acidification (Fig. 1A). Furthermore, the drug did not increase the expression of proautophagic protein beclin-1 or conversion of LC3-I to its lipidated, autophagosome-associated LC3-II form (Fig. 1B). Since the inability of indomethacin to augment LC3-II levels could result from the increase in LC3 degradation in autophagolysosomes [3], we analyzed LC3-II concentration in cells in which autophagic proteolysis was blocked by lysosomal inhibitors chloroquine, bafilomycin A1, or ammonium chloride. While proteolysis inhibitors expectedly increased the levels of LC3-II when applied alone, no additional increase was detected in the presence of indomethacin (Fig. 1C), thus confirming its inability to increase autophagic flux. We next employed genetic and pharmacological approaches to assess the role of autophagy in indomethacin-mediated cytotoxicity to U251 cells [2]. RNA interference-mediated downregulation of autophagy-essential genes LC3 and beclin-1 (the knockdown efficiency confirmed by immunoblot in Fig. 2A) had no effect on the viability of indomethacin-treated U251 cells (Fig. 2B). Accordingly, the suppression of autophagy by proton-pump blocker bafilomycin A1, lysosomal inhibitor chloroquine, or class III phosphoinositide 3-kinase inhibitor wortmannin [3] failed to affect antiglioma effect of indomethacin (Fig. 2C).

## 2 Experimental design, materials and methods

### 2.1 Cells and cell culture

The human glioma cell line U251-MG was obtained from the European Collection of Authenticated Cell Cultures (ECACC 09063001), and maintained in 20 mM HEPES (4-(2-hydroxyethyl)-1-piperazineethanesulfonic acid)-buffered Roswell Park Memorial Institute (RPMI) 1640 cell culture medium supplemented with 2 mM L-glutamine, antibiotic/antimycotic mixture (1%), and 10% fetal bovine serum (all from PAA, Pasching, Austria). U251 glioma cells before passage 10 were incubated in 96-well flat bottom plates (1×10^4^ cells/well) for the cell viability assays, 24-well plates (1×10^5^ cells/well) for the flow cytometry analysis, or 90 mm Petri dishes (2×10^6^ cells) for the immunoblotting. Cells were rested for 24 h and then treated with indomethacin (Sigma-Aldrich, St. Louis, MO) in the presence or absence of bafilomycin A1, chloroquine, ammonium chloride, or wortmannin (all from Sigma-Aldrich, St. Louis, MO). The incubation times and concentrations of agents are stated in figure captions and/or figures.

### 2.2 Determination of cell viability and cell death

The cell viability was determined exactly as previously described, using MTT (3-(4,5-dimethylthiazol-2-yl)-2,5-diphenyltetrazolium bromide) assay to measure the activity of mitochondrial dehydrogenases [4]. The results were presented as % of the cell viability in untreated cells.

### 2.3 Intracellular acidification measurement

The changes in intracellular acidification were assessed by supravital staining with acridine orange (Sigma-Aldrich, St. Louis, MO), a pH-sensitive dye that shifts its fluorescence from green to red with decreasing pH. Acridine orange-stained cells (1 µM, 15 min, 37 °C) were analyzed using FACSCalibur flow cytometer (BD Biosciences, Heidelberg, Germany) and CellQuest Pro software, and intracellular acidification was quantified as a red/green fluorescence ratio (mean FL3/FL1), arbitrarily set to 1 in control samples.

### 2.4 Immunoblotting

Cells were lysed by 30 min incubation on ice in lysis buffer (30 mM Tris–HCl pH 8.0, 150 mM NaCl, 1% NP-40) containing protease/phosphatase inhibitor cocktail (all from Sigma-Aldrich, St. Louis, MO). The cell lysates were centrifuged at 14000 g for 15 min at 4 °C, and the supernatants were collected. Equal protein amounts from each sample were separated by SDS-PAGE and transferred to nitrocellulose membranes (Bio-Rad, Marnes-la-Coquette, France). Following incubation with rabbit antibodies against LC3B, beclin-1, and actin (all from Cell Signaling Technology, Beverly, MA) as primary antibodies, and peroxidase-conjugated goat anti-rabbit IgG (Jackson IP Laboratories, West Grove, PA) as a secondary antibody, specific protein bands were visualized using enhanced chemiluminescence reagents (Amersham Pharmacia Biotech, Piscataway, NJ). The protein levels were quantified by densitometry using ImageJ software (NIH, Bethesda, MD), and expressed relative to actin as a loading control. The results are presented as fold change in signal intensity, which was arbitrarily set to 1 in untreated cells.

### 2.5 RNA interference

The transfection of subconfluent U251 cells with small interfering RNA (siRNA) targeting human beclin-1, LC3B, or scrambled control siRNA (FlexiTube GeneSolution with 4 preselected siRNAs for each gene; Qiagen, Valencia, CA) was performed using Lipofectamine (Invitrogen, Carlsbad, CA) according to the manufacturer׳s instructions. After transfection, cells were grown for 24 h before treatment with indomethacin.

### 2.6 Statistical analysis

The statistical significance of the differences was analyzed by one-way analysis of variance (ANOVA) followed by Student-Newman–Keuls test. A *p* value of less than 0.05 was considered significant. Each experiment was performed in triplicates and the data are presented as mean±SD from at least three independent experiments.
