# Supplementary material for: Data supporting the inability of indomethacin to induce autophagy in U251 glioma cells
Source: Data Brief. 2017 Feb 10;11:225–30. doi: 10.1016/j.dib.2017.02.012 (PMC5320059; doi:10.1016/j.dib.2017.02.012)
Supplement: Supplementary file 1 — Supplementary material [file mmc1.doc]

**Conflict of Interest Form**

Journal: Data in Brief

Article: Data supporting the inability of indomethacin to induce autophagy in U251 glioma cells (DIB-D-16-00948)

Authors: Aleksandar Pantovic, Katarina Arsikin, Milica Kosic, Biljana Ristic, Vladimir Trajkovic,b Ljubica Harhaji-Trajkovic

The authors declare no conflict of interest.
